# Delineating Novel Signature Patterns of Altered Gene Expression in Schizophrenia Using Gene Microarrays

**DOI:** 10.1100/tsw.2001.17

**Published:** 2001-04-04

**Authors:** Karoly Mirnics, Frank A. Middleton, David A. Lewis, Pat Levitt

**Affiliations:** Department of neurobiology, University of Pittsburgh School of Medicine, PA 15261, USA

**Keywords:** schizophrenia, profrontal cortex, synaptic function, neurosecretion, G-proteins, gene microarrays, polygenic disorders, neurodevelopment

## Abstract

Schizophrenia is a complex and devastating brain disorder that affects 1% of the population and ranks as one of the most costly disorders to afflict humans. This disorder typically has its clinical onset in late adolescence or early adulthood, presenting as a constellation of delusions and hallucinations (positive symptoms); decreased motivation, emotional expression, and social interactions (negative symptoms); and impaired learning and memory (cognitive symptoms). The etiology of schizophrenia is unknown, but appears to be multifaceted, with genetic and epigenetic developmental factors all implicated. A convergence of observations from clinical, neuroimaging, and anatomical studies has implicated the dorsal prefrontal cortex as a major locus of alterations in schizophrenia.

How does one investigate the polygenic dimension of such a complex disorder, in which there is likely to be altered gene expression due to both inherited mutations and to individual functional adaptations? This type of analysis, from cancer to heart disease, is being accomplished with the use of cDNA microarrays[8]. Such arrays enable the expression profiling of thousands of genes at one time by making use of DNA spots from libraries of expressed human genes. By labeling the mRNA from postmortem human brain tissue with fluorescent dyes, one can compare the levels of expressed genes between subjects. For the first time, two new studies, published in the journals *Neuron*[9] and *Molecular Psychiatry*[10], successfully used microarrays in a neuropsychiatric disease to measure the expression levels of over 7,800 genes simultaneously between the prefrontal cortex of subjects with schizophrenia and matched controls. Each comparison created an "activated gene (expression) profile". We were able to define specific, complex patterns of altered gene expression and to identify genes that heretofore had not been associated with schizophrenia. The findings suggest that schizophrenia is a disease of basic dysfunction of neuronal communication.

Historically, analysis of altered gene expression in complex diseases was limited technically to investigating one or a few genes at one time, precluding the discovery of diverse expression differences of perhaps hundreds of genes that may provide a signature pattern of the disease. Altered synaptic function had been implicated previously in single gene studies, but the complexity of the disruption and the identity of new candidate genes was not defined[11,12,13,14,15]. In our studies, we utilized a novel data analysis strategy[9], grouping genes encoding proteins that cooperate to perform specific cellular functions, which allowed us to assess consistent changes for over 250 gene groups across subjects with schizophrenia.

The gene group analysis reported in *Neuron*[9] revealed several unexpected findings. First, only a few gene groups were consistently changed between subjects with schizophrenia and matched controls. The most changed gene group, altered in all subjects with schizophrenia, was related to signaling between neurons (presynaptic secretory function = PSYN gene group), suggesting that subjects with schizophrenia share a common abnormality in neurotrans-

* Dr. Pat Levitt, Phone: 412-383-8910, Fax: 412-648-1441, e-mail: plevitt@pitt.edu
© 2001 with author.

mitter release. There were several consistently changed genes within the PSYN gene group. Expression levels of two genes, N-ethylmaleimide-sensitive factor (NSF) and synapsin2 (SYN2) were the most decreased genes. We were able to verify this decrease in the same subjects and a new set of schizophrenic subjects, with a more conventional molecular biology technique, *in situ* hybridization. The consistency and robustness of our findings raise the possibility that NSF and SYN2 expression deficits are directly related to the more conserved symptoms of the disease. The most affected genes in the PSYN group varied from schizophrenic subject to subject, showing distinct "molecular signatures" of schizophrenia in each subject. Schizophrenia, therefore, may involve deficits in different genes in one functional group that lead to the impairment of the same function – signaling between neurons.

In a new, related study from our laboratory, to be published in the May issue of *Molecular Psychiatry*[10], we report that the most consistently changed gene of the over 7,800 analyzed encodes a protein from the regulator of G-protein signaling (RGS) family[16]. Proteins in this family modulate response duration of postsynaptic neurons following the release of presynaptic neurotransmitters that bind to G-protein coupled receptors, such as the metabotropic glutamate, 5-$HT_2$ or $D_2$ dopamine receptors. Expression of the gene RGS4 had never been identified as defective in schizophrenia, but the microarray analysis found this gene to be decreased 50–84% in all 6 subjects examined. These observations were verified by *in situ* hybridization and confirmed in 4 out of 5 additional schizophrenic subjects. Moreover, RGS4 levels were decreased in not only the prefrontal cortex of 9/10 schizophrenic subjects, but also the motor and visual cortices of the same schizophrenic subjects.

Although the precise effects of a reduction in RGS4 are somewhat difficult to predict, it is clear that such a reduction would result in longer duration of postsynaptic signaling in many populations of cortical neurons. In this respect, it is interesting to note that RGS4 has been characterized as a stress-response gene in animal studies[17], where levels of expression change in response to physical stressors. Thus, the cortical RGS4 decrease in schizophrenic subjects could help explain the well-described exacerbation of symptoms by those patients in the face of stress.

In contrast to the RGS4 findings, gene group analysis showed that over 200 genes related to G-protein signaling were unchanged. These observations suggest that the RGS4 expression defect is a specific molecular adaptation, perhaps due either to decreased presynaptic efficiency of neurotransmitter release, or to an inherited mutation. Remarkably, the potential inadequacies in the regulation of G-protein signaling due to RGS4 deficiency is consistent with the therapeutic efficacy of typical and atypical anti-psychotics[19,20,21,22], which target the very Gi/Go and Golf receptors that are regulated by RGS4. In terms of a potential mutational mechanism, it is worthwhile to note that RGS4 maps to chromosomal locus 1q21.22, which was recently reported as a highly significant schizophrenia susceptibility locus[18]. We are currently screening the RGS4 gene for mutations and polymorphisms to formally address this possibility.

In light of these findings, what is our current understanding of schizophrenia? We proposed a model, described in detail in our most recent publications[9,10], suggesting that different genetic insults of genes related to synaptic communication, combined with other factors, may lead to shared clinical manifestations of schizophrenia. First, we believe that the etiology of schizophrenia involves a polygenic pattern of inheritance, resulting in altered function of proteins controlling the 'mechanics' of synaptic transmission. Second, we interpret the heterogeneity in expression defects within the PSYN group as a reflection of distinct adaptive capacities of different populations of neurons. The alterations in RGS4 could reflect a primary gene defect or a postsynaptic adaptation to enhance neurotransmission in light of the defects in PSYN function. Third, deficits in PSYN and RGS4 gene expression may affect the extended postnatal developmental process of synapse formation and pruning, ultimately providing the link to the neurodevelopmental time-course of schizophrenia. Fourth, independent of their roles in causing the disease, the deficits in PSYN and RGS4 expression have physiological and behavioral consequences relevant to the pathophysiology of schizophrenia.

Our discoveries of altered gene expression provide new candidates for mutational analyses, novel pharmacotherapeutics development, and perhaps even the production of independent screening methods to facilitate preclinical diagnosis that ultimately could lead to the prevention of the expressed symptoms of schizophrenia.

## ACKNOWLEDGEMENTS

The research summarized here was supported by projects 1 (DAL) and 2 (PL, KM) of NIMH Center Grant MH45156 (DAL), an endowment fund from the R.K. Mellon Foundation (PL), and NIMH training grant T32 MH18273 and a NARSAD Fellowship (FM).
